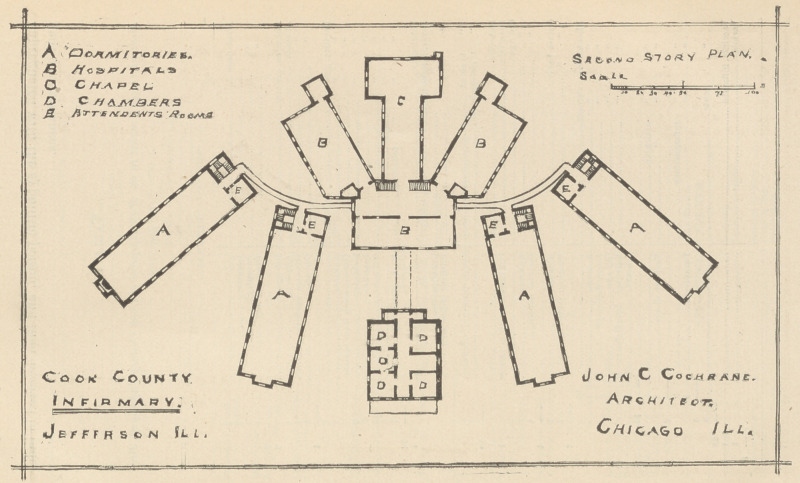# Practice at the County Infirmary

**Published:** 1884-01

**Authors:** A. W. Hagenbach

**Affiliations:** Medical Superintendent


CHICAGO MEDICAL

### JOURNAL & EXAMINER.

VOL. XLVIII.—JANUARY, 1884.—No. 1.

### Original Communications.

### Article I.

PRACTICE AT THE COUNTY INFIRMARY. By A. W. HAGENBACH M.D., Medical Superintendent.

In the March, 1882, number of the JOURNAL AND EXAMINER, was published my first article on "Practice at the County Infirmary," with tables showing the total number of male patients treated in the Hospital Department during the previous year, giving a detailed account of several cases of special interest. During the year 1882, a large number of interesting cases have been admitted, and as this is the second report ever made showing the medical status of the institution, I have prepared a number of tables, embracing Diagnoses; Ages; Cause of Death, etc., of every male patient treated during the year. A great deal of just criticism has been indulged in by the daily press and visiting public concerning the dilapidated condition of the old infirmary buildings. This cause for complaint is happily removed, as we now occupy the new structure, conceded to be one of the best buildings of its kind in the United States. The new buildings will accommodate 800 inmates, including hospital accommodations for 150 patients. The lying-in ward is pleasantly situated, well lighted and heated, and contains 20 beds. The steam heating and ventilating apparatus are of the most approved pattern. The buildings are heated throughout by direct radiation, circulating coils being employed in the large dormitories, twelve in number, and radiators in the general kitchen, dining-rooms, corridors, administration and hospital buildings. The accompanying diagrams (see pp. 4-5) will show the plan of two stories of the new buildings.

### DESCRIPTION OF COOK COUNTY INFIRMARY.

The plan of the building is a departure from similar buildings heretofore erected, and is original with Mr. J. C. Cochrane, the architect, and, as experience teaches, is admirably adapted to charity buildings, affording as it does light and ventilation, and a great amount of sunshine; the plan also commends itself as convenient regarding the domestic and culinary department, the different wings radiating from two common centers, places the inner ends near to the kitchen, laundry and dining-room, thereby saving distance for transportation of food, the greatest distance from the dining-room to the kitchen being 26 feet.

The basement story contains workshops, boiler and engine-room, laundry, office, reception room, etc.

The principal story contains the parlors, dining-rooms, warden's office, doctor's room, dispensary, kitchen, dormitories, sewing and reading-room.

The second story contains warden's private room, hospitals and dormitories; the third story, the same.

The exterior is quite picturesque; the different wings radiating, and the sharp roofs giving a grand effect to the group. The walls are of solid brickwork, and there are verandahs, two and three stories high, affording accommodation for rustication and exercise.

One of the most desirable features of the plan is that the sun will shine in every room in the building twice each day, morning and evening.

Another cause for congratulation is the recognition by the county authorities of primary syphilis as a disease entitled to hospital treatment. In my last article, I called attention to the want of provisions for this unfortunate class of patients, as follows:

"The rules governing the admission of patients to the charitable institutions supported by this county, prohibit the admission of patients suffering with primary syphilis. The city of Chicago has made no provisions whatever for this unfortunate class of patients; cases are not infrequently admitted with secondary, or even tertiary symptoms, who have received no previous treatment." *

This unjust discrimination against syphilis has at last been abandoned; and while I am opposed to the indiscriminate mixing of these patients with the patients of a general hospital ward charity demands that they be admitted to the hospitals, and a ward set aside for their treatment until the authorities awake to the necessity of making suitable separate provisions for their case. The insane, feeble-minded, deaf, blind, and sick are cared for in costly buildings, at the State's, or local government's expense, and I can see no just reason why the syphilitic alone should be excluded from the benefit of the lavish charity bestowed upon all others in distress. I have known of several instances where indigent persons suffering with tertiary syphilis were refused admission to the various charitable institutions of Chicago until the police authorities insisted upon their removal from the public streets on account of the loathsome appearance they presented. The argument I once heard advanced by a person in authority, that syphilis should be regarded as a crime more than a misfortune, would apply with equal force to masturbative insanity, alcoholism, and various other affections that are treated in insane asylums and hospitals. I cannot conceive of a case calling for more sympathy, or entitled to better care at the public expense, than the virtuous wife of a besotted husband who has become

* A case of neglected syphilis was reported by me in the March, 1882, number of the JOURNAL AND EXAMINER.

impregnated with the syphilitic poison, and then deserted in indigent circumstances.

### TABLE I.

*Medical Cases Treated in the Male Hospital of Cook County Infirmary dur- the year* 1882.

GENERAL.

| | | | | | |
|---|---|---|---|---|---|
| Alcoholism | | | 4 | | |
| " | Inanition | | 2 | | |
| " | Paraplegia | | 1 | | |
| " | Ulcers of leg | | 1 | | |
| | | | | 8 | |
| Anasarca, general | Old age | | | 2 | |
| Anæmia | Anasarca | | 1 | | |
| " | General debility | | 1 | | |
| " | Old age | | 2 | | |
| | | | | 4 | |
| Debility, general | | | 2 | | |
| " " | Anæmia | | 1 | | |
| " " | Convalescing from variola | | 1 | | |
| " " | Dementia | | 2 | | |
| " " | Inanition | | 3 | | |
| " " | Old age | | 11 | | |
| " " traumatic | | | 1 | | |
| " " " following | Nerve stretching | | 1 | | |
| | | | | 22 | |
| Febricular | | | | 1 | |
| Fever, intermittent | | 4 | | | |
| " " | Constipation | 1 | | | |
| " " | Phthisis pulmonalis | 1 | | | |
| | | | 6 | | |
| " typhoid | | | 3 | | |
| " typho-malarial | | | 1 | | |
| | | | | 10 | |
| Imbecile | | | | 1 | |
| Inanition | Convalescence from variola | | | 1 | |
| Old Age | | | 2 | | |
| " | Anæmia | | 1 | | |
| " | General debility | | 1 | | |
| | | | | 4 | |
| Opium habit | Alcoholism | | 1 | | |
| " " | Ulcer on vertex of head | | 1 | | |
| | | | | 2 | |
| Rheumatism, chronic | | 13 | | | |
| " " | Anæmia | 1 | | | |
| " " | Joints deformed | 2 | | | |
| " " | Old age | 1 | | | |
| " " | Valvular insufficiency of heart | 2 | | | |
| | | | 19 | | |
| " " articular | | 1 | | | |
| " " " | Varicose ulcers | 1 | | | |
| | | | 2 | | |
| " " inflammatory | | 3 | | | |
| " " " | Paraplegia | 1 | | | |
| | | | 4 | | |
| " acute | | | 3 | | |
| | | | | 28 | |
| Vaccinia, inflamed | | | | 1 | |
| Variola | | | | 1 | |
| | | | | | 85 |

GASTRO-INTESTINAL.

| | | | | | |
|---|---|---|---|---|---|
| Diarrhœa, acute | | 1 | | | |
| " " | Secondary syphilis | 1 | | | |
| | | | 2 | | |
| " chronic | | 19 | | | |
| " " | Anæmia | 2 | | | |
| " " | Bronchitis | 1 | | | |
| " " | Caries of elbow | 1 | | | |
| " " | General debility | 5 | | | |
| " " | Dementia | 4 | | | |
| " " | Febricula | 1 | | | |
| " " | Hemiplegia, right | 1 | | | |
| " " | Hemorrhoids | 1 | | | |
| " " | Hernia, double inguinal | 1 | | | |
| " " | Inanition | 1 | | | |
| " " | Old age | 2 | | | |
| " " | Chronic rheumatism | 1 | | | |
| | | | 40 | | |
| | | | | 42 | |
| Dysentery, acute | | | 2 | | |
| " chronic | | | 2 | | |
| | | | | 4 | |
| Enteralgia | | | | 1 | |
| Enteritis | | | 1 | | |
| " chronic | | | 1 | | |
| | | | | 2 | |
| Gastritis, sub-acute | Diarrhœa | | | 1 | |
| Gastro-enteritis | | | 1 | | |
| " " | Bronchitis | | 1 | | |
| " " | Diarrhœa | | 3 | | |
| " " | Icterus | | 2 | | |
| | | | | 7 | |
| Morbus plumbeus | | | | 1 | |
| | | | | | 58 |

### TABLE II.

*Surgical Cases Treated in the Male Hospital of Cook County Infirmary, During the year* 1882.

AFFECTIONS OF BONES AND APPENDAGES.

| | | | | |
|---|---|---|---|---|
| Ankle enlargement, chronic | Anæmia | | 1 | |
| Bone hypertrophy | | | 1 | |
| Caries, tarsus | | 1 | | |
| " elbow joint | | 2 | | |
| | | | 3 | |
| Dislocation, wrist | | | 1 | |
| Fracture, acromion process | | 1 | | |
| " femur | | 1 | | |
| " radius | | 1 | | |
| " ribs | | 1 | | |
| " tibia and fibula | | 2 | | |
| | | | 6 | |
| Injury to provisional callus | | | 1 | |
| Knee joint enlarged, traumatic | | | 1 | |
| Leg deformed, traumatic | | | 1 | |
| Sprain, ankle | | | 1 | |
| Spinal injury | | | 2 | |
| Toe, deformed | | 1 | | |
| " crushed | | 1 | 2 | |
| | | | | 20 |

AFFECTIONS OF THE EYE.

| | | | |
|---|---|---|---|
| Amaurosis | Anæmia | 1 | |
| " | General debility | 2 | |
| | | | 3 |
| Cataract senile | General debility | | 2 |
| Conjunctivitis | Ulcer of leg | 1 | |
| " chronic | Bronchitis | 1 | |
| | | | 2 |

| | | | | |
|---|---|---|---|---|
| Hypopyon | | 1 | | |
| " | Imbecile | 1 | | |
| | | | 2 | |
| Lens crystalline, displaced | | | 1 | |
| Ophthalmia, gonorrhœal | | | 1 | |
| | | | | 11 |

AFFECTIONS OF INTEGUMENT.

| | | | | |
|---|---|---|---|---|
| Combustivo derma | | | 2 | |
| Eczema | | 1 | | |
| " chronic | Indolent ulcer of leg | 1 | | |
| | | | 2 | |
| Erysipelas, face | | 1 | | |
| " " and head | | 1 | | |
| | | | 2 | |
| Herpes, diffus. syphilitic | | | 1 | |
| Scabies | | | 2 | |
| | | | | 9 |

SPECIFIC.

| | | | | |
|---|---|---|---|---|
| Cancer, pylorus | | | 1 | |
| Scirrhus, tongue | | 1 | | |
| " face | | 1 | | |
| | | | 2 | |
| Syphilis, hereditary | | 1 | | |
| " " | Ulcers face and neck | 1 | | |
| | | | 2 | |
| " primary | Chancroidal ulcers | 1 | | |
| " " | Gonorrhœal | 1 | | |
| " " | Suppurating bubo | 1 | | |
| | | | 3 | |
| " secondary | | 1 | | |
| " " | Anæmia | 1 | | |
| " " | Suppurating bubo | 1 | | |
| " " | Ulcers, pharynx | 1 | | |
| | | | 4 | |
| " tertiary | | 1 | | |
| " " | Destruction soft palate | 1 | | |
| | | | 2 | |
| | | | | 14 |

GENERAL.

| | | | | |
|---|---|---|---|---|
| Abscess, leg | Erysipelas | 2 | | |
| " nates | | 1 | | |
| | | | 3 | |
| Apoplexy, cerebral | | 1 | | |
| " " | Partial hemiplegia | 1 | | |
| " " | Right " | 1 | | |
| | | | 3 | |
| Anthrax, leg | | | 1 | |
| Amputation, arm | | 1 | | |
| " leg | | 1 | | |
| | | | 2 | |
| Bruises | | | 1 | |
| Cystitis | | 1 | | |
| " | Chronic rheumatism | 1 | | |
| | | | 2 | |
| Cerebral concussion | | | 1 | |
| Ecchymosis, traumatic | Dementia | | 1 | |
| Epistaxis | | | 1 | |
| Fistula, urethra | | | 2 | |
| Frost bite, feet | | 1 | | |
| " " | Dementia | 1 | | |
| " " | Melancholia | 1 | | |
| " fingers and toes | | 1 | | |
| | | | 4 | |
| Hernia, double inguinal | | 1 | | |
| " strangulated, oblique inguinal | | I | | |
| | | | 2 | |
| Lymphadenitis, suppurating | | | 1 | |
| Neurotrosis | | | 1 | |
| Orchitis, acute | | | 1 | |

| | | | | |
|---|---|---|---|---|
| **Paralysis, traumatic** | | | 1 | |
| **Paraplegia,** " | | | 1 | |
| **Prolapsus rectum** | Dementia | | 1 | |
| **Strain quadratus lumborum** | | | 1 | |
| **Stricture, urethra** | | | 2 | |
| **Synovitis, right knee** | | 2 | | |
| " left " | | 3 | | |
| | | | 5 | |
| **Ulcers, chancroidal** | Phymosis | 2 | | |
| " " | Suppurating bubo | 1 | | |
| | | | 3 | |
| " cornea | Trichiasis | | 1 | |
| " in cicatrix | | | 1 | |
| " gastric | | | 1 | |
| " heel traumatic | | | I | |
| " inflamed leg | | | 1 | |
| " indolent leg | | 2 | | |
| " " " | Chronic marla | 1 | | |
| | | | 3 | |
| " over malleolus | Hypertrophy of leg | | 1 | |
| " phagedenic | Dementia | | 1 | |
| " inf. ext. syphilitic | | | 1 | |
| " tonsils | Febricula | 1 | | |
| " varicose | | 3 | | |
| " " | Rheumatism | 1 | | |
| | | | 4 | |
| " syphilitic | | | 1 | |
| | | | | 20 |
| **Vesicles, foot, traumatic** | | | 1 | |
| **Wound, elbow** | | | 1 | |
| " foot, penetrating | | | 1 | |
| " gunshot | | | 1 | |
| " incised | | | 1 | |
| " knee, penetrating | | | 1 | |
| " lacerated | | | 2 | |
| " scalp | | | 1 | |
| | | | 8 | 66 |
| Total | | | | 120 |

HEART.

| | | | | |
|---|---|---|---|---|
| **Angina pectoris** | Bronchitis | | 1 | |
| **Endo-carditis** | Infl. rheumatism | | 1 | |
| **Valvular lesions of heart** | | 2 | | |
| " " | General anasarca | 2 | | |
| " " | Enlargement by dilatation | 2 | 6 | |
| | | | | 8 |

KIDNEYS.

| | | | |
|---|---|---|---|
| **Albuminuria** | | 5 | |
| " | General anasarca | 2 | |
| " | Bronchitis | 1 | |
| " | Hernia, left inguinal | 2 | |
| " | Peritoneal | 1 | |
| | | | 11 |

LUNGS, ETC.

| | | | |
|---|---|---|---|
| **Asthma with bronchitis** | | 9 | |
| " " | Hernia hypogastrium | 1 | |
| | | | 10 |
| **Bronchitis** | | 7 | |
| " | Anæmia | 6 | |
| " | General debility | 5 | |
| " | Diarrhœa | 1 | |
| " | Dorsal curvature, lateral | 1 | |
| " | Hypertrophy of heart | 1 | |
| " | Valvular lesions of heart | 1 | |
| " | Old age | 4 | |
| " | Paralysis of arm, traumatic | 1 | |
| | | | 27 |
| **Emphysema pulmonum** | Diarrhœa | | 1 |
| **Laryngitis** | | | 1 |

| | | | | |
|---|---|---|---|---|
| Phthisis pulmonalis | | 17 | | |
| " " | Anæmia | 1 | | |
| " " | Aphonia | 1 | | |
| " " | Bronchitis | 4 | | |
| " " | Convalescing from variola | 1 | | |
| " " | Chest contracted | 1 | | |
| " " | Hemorrhoids | 1 | | |
| " " | Icterus | 1 | | |
| " " | Inanition | 1 | | |
| " " | Pneumonia, circumscribed | 1 | | |
| " " | Icterus | 1 | | |
| " " | Tubercular laryngitis | 2 | | |
| " " | Valvular lesions of heart | 2 | | |
| | | | 34 | |
| Pleuritis circnmscribed | | 2 | | |
| " left side | Ptosis right eye-lid | 1 | | |
| | | | 2 | |
| Pleuro-pneumonitis | | 1 | | |
| " " | Valvular insufficiency | 1 | | |
| | | | 2 | |
| Pneumonitis | | 1 | | |
| " circumscribed | | 2 | | |
| " | Alcoholism | 1 | | |
| " | Bronchitis | 1 | 5 | |
| | | | | 83 |

NERVOUS SYSTEM.

| | | | | |
|---|---|---|---|---|
| Aphasia | Hemiplegia, partial | | 1 | |
| Cardiagra | | | I | |
| Chorea | | 2 | | |
| " chronic | Anasarca, general | 1 | | |
| | | | 3 | |
| Dementia | | 1 | | |
| " | Hemplegia | 1 | | |
| " | Old age | 4 | | |
| | | | 6 | |
| Epilepsy | Anæmia | 2 | | |
| " | Bronchitis | 1 | | |
| " | Hemiplegia | 1 | | |
| " | Paraplegia, partial | 1 | | |
| | | | 5 | |
| Hemiplegia, right | | 2 | | |
| " " | Complete aphasia | 1 | | |
| | | | 3 | |
| " left | | 2 | | |
| " " | Cephalalgia, frontal | 1 | | |
| " " | Dementia | 1 | | |
| " " | Fracture, neck of left femur | 1 | 5 | |
| | | | | 8 |
| Hyperæmia, spinal cord | Partial paralysis | | | 1 |
| Mania, chronic | | | 1 | |
| " " | Paralysis of extremities | | 1 | |
| " recurrent | | | 1 | |
| " sub-acute | Nasal catarrh | | 1 | |
| | | | | 4 |
| Melancholia s | Hysteria | 1 | | |
| " | Chronic rheumatism | 1 | | |
| " sub-acute | | 1 | | |
| " suicidal | | 1 | | |
| | | | 4 | |
| Neuralgia, facial | | 1 | | |
| " intercostal | | 1 | | |
| | | | 2 | |
| Neurasthenia | | | 1 | |
| Paralysis, agitans | Hernia, inguinal | 1 | | |
| " extremities, complete | Dementia | 1 | | |
| " partial | Mania, chronic | 1 | | |
| " rectum and bladder | | 1 | | |
| | | | 4 | |
| Paraplegia | Paralysis, rectum and bladder | | 2 | |
| Pleurodynia | | | 1 | |
| Spinal sclerosis | Hemiplegia, right | | 1 | |
| Tabes dorsalis | | 2 | | |
| " " | General debility | 2 | | |
| " " | Orchitis, right testicle | 1 | | |
| " " | Ulcers, floor of mouth | 1 | 6 | 50 |
| | Total | | | 295 |

### TABLE III.

*Nativity of Male Patients Treated in Hospital During the Year* 1882.

| | | | |
|---|---|---|---|
| United States, white | 74 | Holland | 2 |
| " " colored | 9 | Hungary | 1 |
| | 83 | Ireland | 120 |
| Belgium | 1 | Norway | 18 |
| Bohemia | 8 | Poland | 3 |
| Canada | 3 | Russia | 1 |
| Denmark | 7 | Scotland | 16 |
| East Indies | 1 | Sweden | 24 |
| England | 15 | Switzerland | 1 |
| France | 2 | | |
| Germany | 108 | Total | 415 |

### TABLE IV.

*Age of Male Patients Treated in Hospital During the Year* 1882.

| | | | |
|---|---|---|---|
| Under 20 | 9 | From 60 to 65 | 36 |
| From 20 to 25 | 19 | " 65 " 70 | 49 |
| " 25 " 30 | 23 | " 70 " 75 | 33 |
| " 30 " 35 | 21 | " 75 " 80 | 19 |
| " 35 " 40 | 34 | " 80 " 85 | 25 |
| " 40 " 45 | 25 | " 85 " 90 | 5 |
| " 45 " 50 | 36 | " 90 " 95 | 1 |
| " 50 " 55 | 38 | | |
| " 55 " 60 | 42 | Total | 415 |

### TABLE VI.

*Nativity of Patients who have Died in the Male Hospital of Cook County Infirmary During the Year* 1882.

| | | | |
|---|---|---|---|
| United States, white | 17 | Holland | 1 |
| " " colored | 3 | Ireland | 31 |
| | 20 | Norway | 4 |
| Belgium | 1 | Scotland | 1 |
| Bohemia | 2 | Sweden | 9 |
| Canada | 1 | | |
| England | 2 | Total | 110 |
| Germany | 38 | | |

### TABLE VII.

*Age of Patients who have Died in the Male Hospital of Cook County Infirmary During the Year* 1882.

| | | | |
|---|---|---|---|
| From 20 to 25 | 3 | From 60 to 65 | 13 |
| " 25 " 30 | 5 | " 65 " 70 | 10 |
| " 30 " 35 | 3 | " 70 " 75 | 13 |
| " 35 " 40 | 5 | " 75 " 80 | 11 |
| " 40 " 45 | 8 | " 80 " 85 | 9 |
| " 45 " 50 | 6 | " 85 " 90 | 2 |
| " 50 " 55 | 10 | | |
| " 55 " 60 | 12 | Total | 110 |

### TABLE V.

*Cause of Death of Patients in Male Hospital of Cook County Infirmary during the year* 1882.

| *Immediate Cause.* | *Complications.* | | |
|---|---|---|---|
| Albuminuria | | 1 | |
| " | Anasarca | 2 | |
| " | Coma | 1 | |
| " | Phthisis | 1 | |
| | | — | 5 |
| Anasarca | Old age | 1 | |
| " | Valvular insufficicncy of heart | 1 | |
| | | — | 2 |
| Anæmia | Old age | | 4 |
| Apoplexy, cerebral | Anæmia | 1 | |
| " " | Asthma | 1 | |
| " " | Diarrhœa | 1 | |
| " " | Hemiplegia | 1 | |
| " " | Imbecile | 1 | |
| " " | Old age | 1 | |
| " " | Paralysis rectum and bladder | 1 | |
| " " | Phthisis | 1 | |
| " " | Valvular insufficiency of heart | 1 | |
| | | — | 9 |
| Asthma and bronchitis | | | 3 |
| Bronchitis | Diarrhœa | 2 | |
| " | Inanition | 1 | |
| " | Old age | 2 | |
| | | — | 5 |
| Chorea | Debility | | 1 |
| Cystitis | Rheumatism | | 2 |
| Debility, general | | 1 | |
| " " | Bronchitis | 1 | |
| " " | Inanition | 1 | |
| " " | Old age | 5 | |
| " " | Tertiary syphilis | 1 | |
| | | — | 9 |
| Dementia | Old age | 1 | |
| " | Paralysis | 2 | |
| " | " rectum | 1 | |
| | | — | 4 |
| Diarrhœa | | 4 | |
| " | Bronchitis | 1 | |
| " | Caries of elbow | 1 | |
| " | Dementia | 2 | |
| " | Gastro-enteritis | 1 | |
| " | Old age | 3 | |
| " | Ulcers | 1 | |
| | | — | 13 |
| Dysentery | | | 5 |
| Enteritis | | | 1 |
| Erysipelas | Embolism | | 1 |
| Fever, remittent | Anæmia | 1 | |
| " typhoid | Pulmonary congestion | 1 | |
| | | — | 2 |
| Gastro enteritis | Icterus | 1 | |
| " " | Inanition | 1 | |
| " " | Old age | 1 | |
| | | — | 3 |
| Heart, valvular lesions | Anasarca | 2 | |
| " " " | Enlargement of heart by dilatation | 1 | |
| | | — | 3 |
| Hemiplegia | Cerebral apoplexy | | 1 |
| Inanition | | | 1 |
| Marasmus senile | Debility | | 1 |
| Melancholia | Hysteria | | 1 |
| Opium habit | Peritonæal dropsy | | 1 |
| Paralysis, partial | Debility | | 1 |
| Phthisis pulmonalis | | 10 | |
| " " | Anasarca | 1 | |
| " " | Anæmia | 1 | |
| " " | Diarrhœa | 1 | |
| " " | Valvular lesions of heart | 1 | |
| " " | Laryngitis | 1 | |
| " " | Pneumonia, circumscribed | 2 | |
| " " | Pulmonary hæmorrhage | 1 | |
| | | — | 18 |

| | | | |
|---|---|---|---|
| Pleuritis, double | Pneumonia, lobular | | 1 |
| Rheumatism | Anæmia | 1 | |
| " | Old age | 1 | |
| " | Ulcers | 2 | |
| | | — | 4 |
| Rigor congestiv | Icterus | | 1 |
| Scirrhus stomach | | 1 | |
| " " | Hæmatemesis | 1 | |
| " tongue | | 1 | |
| | | — | 3 |
| Stasis epilepticus | Hemiplegia | | 1 |
| Synovitis | Cirrhosis of liver | | 1 |
| Tabes dorsalis | Abscess, floor of mouth | 1 | |
| " " | Debility | 1 | |
| " " | Old age | 1 | |
| | | — | 3 |
| | Total | | — 110 |

The tables show that 295 medical, and 120 surgical cases were admitted to male hospital department during the year. The 8 cases of alcoholism were mostly former inmates who were refused admission to the County Hospital, Chicago, being regarded as infirmary patients. The 26 cases of anæmia, and general debility, were mostly old persons, or convalescents from variola, surgical operations, etc., the anæmia or debility being the principal feature in the cases requiring treatment. Of the 7 cases of malarial fevers, but one originated on the premises. The infirmary buildings are located on a dry, sandy ridge, and the inmates are remarkably free from malaria. Two of the three typhoid fever patients were recent European immigrants. The imbecile was admitted for a short time to hospital department during the extreme cold weather, as he had a tendency to stray away. The 4 cases entered as "old age" were sent to hospital department, on account of their very helpless condition.

The twenty-five cases of chronic rheumatism include articular, muscular, and syphilitic varieties.

Diseases of the gastro-intestinal tract form a sixth of all the medical cases. Diarrhœa is a very common ailment.

A large percentage of the inmates, previous to their admission go for weeks without a warm meal or proper food of any kind, spending what little money they earn, or can beg, on alcoholic stimulants, living on free lunches composed frequently of stale bread and decomposing meats, causing intestinal irritation, and, not infrequently, gastro enteritis. The cases that originate among the inmates can usually be traced to an insufficient change of diet. A simple cathartic, followed by special diet for a few days, is the best treatment, and generally results in a speedy cure. The six cases entered as "valvular lesions" were patients in whom the insufficiency was so great as to prevent them from living in the general wards.

Next to diarrhœa and phthisis, bronchitis has been the most prevalent disease. The physical signs of some of the very chronic cases, usually encountered in infirmaries, are so marked that the râles can be heard at a distance of several feet from the patient. The treatment from which I have derived the best results in these distressing cases has been the administration of potassium iodide in from five to eight grain doses, with Brown's Mixture or some similar cough preparation, and the application of croton oil to the chest. The latter, if persisted in, will do more towards affording relief than anything I have ever made use of. The usual way of applying the croton oil in these cases is as follows: First, a thorough application to anterior chest walls, making a second or even a third application if vesicles do not appear at the end of twelve hours. When the vesicles begin to dry up and disappear, a coat is applied over posterior chest, and later over lateral chest walls. The entire process is repeated at intervals of a few days until relief is experienced, provided always that the general health of the patient warrants a continuation of the treatment. No other treatment has been followed by as good results, and I am firmly convinced that counter-irritation is one of the most important factors in the successful treatment of chronic bronchitis.

Of the 34 cases of phthisis pulmonalis admitted, a majority were transferred from various city hospitals in an advanced stage of the disease, and presented no unusual features. The expectoration of blood is usually the most alarming symptom, in the estimation of the patients; a number living in constant dread of a fatal hæmorrhage, and frequently feel greatly relieved when assured that but a small percentage of cases terminate in that manner. The great infrequency of deaths occurring during a pulmonary hæmorrhage, is illustrated by the fact that of the hundreds of consumptives that came under my immediate care in this institution during the past seven years, not more than five or six cases have terminated in that manner. Such deaths are painful to witness, the patient being conscious of his condition within a few moments of his death. Two cases of pleurisy, 2 of pleuro-pneumonia, and 5 of pneumonia were admitted. Two of the cases of pneumonia were circumscribed, and limited to the upper lobe of one lung. Under the heading "Nervous System" are included one-sixth of the total number of medical cases treated. The large number of insane cases is explained by the fact that harmless chronic insane patients are sometimes transferred from the Insane Asylum to the Infirmary, where acute symptoms may at any time develop, necessitating their removal to the hospital department for a short period, when, if they do not improve, they are returned to the asylum.

The six cases of tabes dorsalis were sent to hospital, because they were unable to live with comfort in the general wards.

The surgical tables show that 120 cases were treated during the year—20 cases of affections of the bones and appendages, 11 of the eye, 9 of the skin, 14 specific, and 66 unclassified.

Of the affections of the bones, the case of traumatic deformity of the leg is reported further on. The fractures include a fracture of the acromion process—femur, radius, ribs, tibia, and fibula. Nearly all the above were indigent persons, meeting with accidents in neighboring towns. The only eye affection calling for special notice is the case of hypopyon, and a case of gonorrhœal ophthalmia; the former because a prolapsus of the iris took place through an incision for the removal of the pus; the latter on account of the acute character of the attack, both eyes being totally destroyed a short time previous to his admission.

Of the affections of the skin, the case of diffuse herpes is of interest, from the fact that the eruption extended over the entire body, reappearing several times at intervals of a few weeks. The patient had had syphilis, and specific treatment was followed by immediate improvement.

Several cases of scabies are admitted nearly every year. The treatment generally followed is as follows: First, destruction of all wearing apparel; second, general bath; third, daily carbolic acid baths, and application of carbolic acid or sulphur ointments. The 14 cases of syphilis recorded do not include one-half of the cases treated, but only such patients as could not be treated as dispensary cases.

The two operations recorded in tables were performed away from the institution, and sent here for subsequent treatment. The amputations here include an amputation of the leg, on account of cancer of the foot; a re-amputation of leg—railroad accident; amputation of five fingers—frost bite; amputation of left arm, upper third, for deformity; and an amputation of foot following frost bite. As usual, ulcers form the largest percentage of surgical cases, including chancroidal, corneal, gastric, inflamed, indolent, phagedenic, syphilitic, and varicose varieties. The wounds include penetrating, gunshot, incised, lacerated, and contused wounds.

Table V., showing "cause of death," shows that disease of the lungs caused the greatest number of deaths, comprising nearly a fourth of the total number. Eighteen deaths were due to phthisis pulmonalis, 8 to bronchitis or asthma, and 1 to double pleurisy. Twenty-one deaths were due to diseases of the nervous system, as follows: Cerebral apoplexy 9; dementia 4; tabes dorsalis 3; hemiplegia, stasus epilepticus, paralysis, melancholia, and chorea, each one. Seventeen deaths were due to gastro-intestinal troubles. Diarrhoea 13; gastro-enteritis 3; enteritis 1. Diseases of the heart caused three deaths, and specific (all cancers) three more. It will be noticed that not a single death is ascribed directly to syphilis. Thanks to the more correct theories and treatment, syphilis is no longer frequently fatal *per se*. There can be no question, however, that through it the general health is frequently impaired to such an extent as to render the system more susceptible to certain acute attacks of disease, and less able to overcome them. Syphilitic patients succumb to affections that would have made but a slight impression had they been in a normal condition. Syphilis is one of the most encouraging diseases to treat, so far as improvement is concerned, but, unfortunately, we can hope for but little more. Constant treatment is the price of comparative good health. Old men are frequently admitted suffering from syphilitic rheumatism, or some other syphilitic affection, who, when asked if they ever had syphilis, will reply, "Yes; but that was many years ago," and volunteering the information that they were treated in time, and entirely cured, etc., never imagining for a moment that their present ailment is in any way related to their youthful indiscretions. From my experience in the treatment of neglected syphilis, I am very hopeful of improvement, even in the most aggravated cases. A case illustrating this point will be briefly reported.

CASE.—A. W. contracted syphilis about twelve years ago; she led a very dissipated life, and neglected treatment except during a few short periods ever since. When admitted about a year ago, the soft palate was almost completely destroyed; syphilitic nodes had formed on various parts of the body, especially the extremities. The turbinated bones had been discharged through the nostrils and mouth, and several small pieces of dead bone had also separated from skull. When admitted, a bluish swelling about the size of a hen's egg and hard to the touch, was noticed in central frontal region, about an inch above nasal eminence. The surface of the swelling commenced to ulcerate, when dead bone was detected by means of a probe. Under specific and tonic treatment her health improved rapidly. The only symptom experienced by the patient was periodical headaches, and later slight continuous frontal headache, regarded as due to a low grade of meningeal inflammation. It was decided to continue the treatment until the bone had completely separated, as it was feared that the necrosis involved the entire thickness of skull, and that adhesions had taken place between its inner surface and dura mater. She insisted upon an operation, however, trying to loosen and remove the bone herself. An operation being decided upon, she was anæsthetized, and the bone carefully loosened from dura mater, to which it was firmly adherent near its lower end. A pair of forceps and handle of scalpel were employed to break up the adhesions. The piece of dead bone removed involved entire thickness of skull, and measured $1\frac{5}{8}$ by $\frac{7}{8}$ inches. The dura mater was thickened and very vascular. After the bleeding had ceased it could be seen at the bottom of the wound, rising and falling with each pulsation of the brain. No unpleasant symptoms followed until the fourth day after the operation, when she suddenly became restless and had an attack of acute mania, with rapid, bounding pulse, flushed face, and considerable febrile disturbance. She became so violent that she had to be removed from the hospital ward and confined in a cell reserved for insane patients. Potassium bromide, chloral hydrate and fluid extract of ergot were administered with great benefit; all the acute symptoms disappearing at the end of twelve hours. Less acute attacks were experienced at intervals of from five to twenty-four hours, and as the facilities were insufficient for a protracted treatment she was declared insane, and transferred to the Hospital for the Insane, June 7, 1883. June 18, I saw the patient engaged in general house work at the asylum. She spoke quite rationally and expressed herself as feeling relieved. The wound on her forehead had almost closed by granulation. She remained in about the same condition until July 19, when she was re-transferred to the infirmary, where she left of her own accord July 24, with the wound healed, and mentally sound.

SILENT PLEURISY.

An interesting case of silent pleurisy, entered in tables as "Hydrothorax," was admitted March 17, 1883, with the following history:

Æt. 51 years; has always been temperate. The first symptom noticed by himself was pain in lower portion of chest, accompanied by slight cough, in July, 1882. Never had a chill, but coughed considerably throughout. He employed various physicians without deriving any apparent benefit; was admitted to Cook County Hospital, Chicago, but left of his own accord unimproved at the end of three weeks; was re-admitted to hospital Jan. 3, 1883, and discharged unimproved. A few days previous to his admission here, March 17, 1 telephoned to the hospital concerning his case, and was informed that the diagnosis in "hospital register" was "Bright's Disease." When first admitted here he suffered greatly from difficulty in breathing; could not possibly retain the recumbent position. He informed me that he had been in about the the same condition for several months previous to his admission. His lips were of a bluish color, showing improper oxidation; pulse feeble and rapid, with constant cough and but slight expectoration. Upon physical examination his right chest was found by measurement to be one and a half ($\frac{1}{2}$) inches larger than the left, with complete flatness over entire right side. No respiratory sounds could be detected, excepting a few harsh sounds over apex. Over the left lung increased respiratory murmur with a few mucous râles were heard. The case was diagnosticated as hydrothorax. A hypodermic needle was introduced into the right pleural cavity, between seventh and eighth ribs, several inches anterior to their angles, when fluid escaped freely. Not having an aspirator at hand, a small sized canula and trocar was introduced, and thirty (30) ounces of straw-colored liquid withdrawn. The breathing, color of lips and heart action all were improved, affording great relief. The liquid coagulated almost completely with nitric acid. The liquid began to re-accumulate, and all the symptoms gradually grew worse until April 1, when he was tapped as before, and twenty-eight (28) ounces similar fluid was withdrawn, followed by immediate relief. The following day the patient was quite feverish, and remained ill until the 8th, when eight ounces dark-colored fluid was withdrawn. The relief was instantaneous and more permanent than before, the fluid accumulating less rapidly. It did not become necessary to tap him again until May 15, when an aspirator was employed, and eighteen (18) ounces clear serum withdrawn. From the last date he improved steadily, no fluid, necessitating an operation, accumulating thereafter.

June 26. Patient is able to walk half a mile without fatigue. The physical signs at present are dullness over entire right side, due, possibly, to the partial expansion of the air vesicles, and the accumulation of fibrin on the costal and pulmonary pleura. Mucous râles are heard over entire right side. His general health is improving rapidly; has gained several pounds in weight; eats and sleeps much better than formerly. The treatment consisted in good nourishment and tonics, and later, out-door exercise. As for the diagnosis of Bright's Disease, at the County Hospital, I would state that I frequently examined his urine with nitric acid and heat tests for albumen, and under the microscope for tube casts or other evidences of renal disease, but failed in every instance to discover any evidence of disease of the kidney. While in the hospital, he informed me he was under homœopathic treatment. He was discharged cured, July 2.

LIGATION OF FEMORAL AND EXTERNAL ILIAC ARTERIES.

J. M., aged 21 years; had syphilis three years ago; gonorhœa four weeks ago, and suffering, when admitted, from acute orchitis. On October 22, 1882, while attempting to alight from a freight train while in motion, he sustained a compound comminuted fracture of left tibia and fibula, necessitating an amputation of the leg, upper portion of middle third. Dr. D. B. Fonda, of Jefferson, performed the amputation, making a long posterior flap. The patient was then sent to the infirmary for subsequent treatment, eight hours after the accident. Upon examination, after admission, he was found poorly nourished, and somewhat exsanguinated. The flaps had been brought into close apposition with a large number of interrupted sutures. Failing to find the ends of any ligatures externally, I concluded that they had in some way drawn within the flaps during his removal, or some absorbable material used as ligatures and cut off close to the ligated vessels. Hot water applications were directed to be applied continuously. The temperature was normal; pulse slightly accelerated.

Oct. 26. Stump swollen and inflamed. Evidence of accumulation of pus in stump being present, several sutures were divided, when the flaps separated widely, and considerable poorly-conditioned pus escaped. Drainage tubes were inserted, and the hot-water dressings continued.

Nov. 9. The stump is daily growing worse; fully one-half of posterior flap has become gangrenous. The remaining sutures have been divided, and the flaps are supported by means of strips of adhesive plaster. The ends of both bones are protruding, and a large bundle of silk ligatures, cut closely to the arteries ligated, is seen at bottom of wound, accounting for their absence externally at the first examination. I left the institution on a ten days' vacation on this date, leaving Dr. Morgan, of the Cook County Hospital, in charge. As soon as the line of demarcation was established, and not sufficient tissue remaining to cover the ends of the bones, he cut a fresh flap out of healthy tissue, and removed about an inch and a half of the bones Nov. 16. On my return to the institution, Nov. 20, the patient was doing well. He improved until Nov. 23, when, at 1:40 P.M., a free secondary hæmorrhage took place from stump; styptics and pressure controlled the bleeding. He lost about two ounces of blood.

Nov. 24. At 8:15 this P. M. had a second and more profuse hæmorrhage; separated the flaps, and found the anterior tibial artery bleeding per saltum. After several ineffectual efforts to ligate the bleeding vessel in stump, it was decided to ligate femoral in Scarpa's triangle. Several ligatures were first placed around anterior tibial, but owing to the degenerate condition of its coats, they invariably cut completely through the vessel. With the assistance of Dr. Alex. Theummler, the femoral was ligated about five inches below Poupart's ligament. A vein of considerable size, lying in front of the artery, was crowded to one side. The patient recovered readily from the shock of the operation, and in a few days showed marked signs of improvement, in increased circulation of blood in lips, improvement of appetite, sleep, and spirits.

Dec. 4. The ligature came away while changing dressings this morning. The patient is improving steadily.

Dec. 7. In passing through ward to-day found the patient sitting up in bed. He was whistling and feeling in the very best of spirits. I cautioned him about the danger of overexertion causing a hæmorrhage from femoral.

Dec. 9. At 11:20 this P. M. had a profuse hæmorrhage from femoral at seat of ligature. The hospital nurse, acting under previous instructions, wound a rubber bandage as tightly as possible above seat of hæmorrhage, completely arresting it. After a consultation with Drs. Spray and Theummler, and with their assisance, ligated the external iliac artery. After the patient was anæsthetized, carried an incision about three and a half inches in length, an inch above and parallel with Poupart's ligament. After the primary incision the transversalis fascia was divided upon a grooved director. Pushing the peritoneum to one side the pulsation of the artery could be plainly felt. The vein was to the inner side and slightly overlapping the artery. After separating the two with the handle of a scalpel, the aneurism needle was passed around the vessel from without inwards, instead of the reverse, as usually recommended. The bifurcation of the common into the external and internal iliac arteries could be plainly felt by passing the finger along the ligated artery. The ligature was applied about midway between the origin of the artery and Poupart's ligament, to afford the greatest chance for a firm coagulum to form in either extremity of the vessel. The operation was performed by the partial antiseptic method.

Dec. 13. Had two chills this A. M., followed by high fever.

Dec. 15. Had one chill to-day. Pulse small and rapid.

Dec. 16. Pulse 112. Temperature 100°.

Dec. 17. Pulse 108; temperature 99. Feels better. Takes nourishment freely and with relish.

Dec. 18. Pulse 96; temperature 99.

Dec. 19. Had a chill to-day, followed by high fever. Pulse 156. Administered stimulant, freely, when pulse began to fall to 90 at 6 P. M.

December 21. Pulse 96. Stump pale and smooth.

December 26. Pulse 108. Appearance of stump about the same.

December 29. Pulse 120, and feeble. Temp. 102½°. Anterior border of tibia ulcerated through stum. Patient was delirious during the night. Small bed-sore has formed over central sacral region.

December 30. Very low, this P. M. Pulse 120, very feeble. Stump pale and cold.

Died at 10:25 this P. M. The general treatment consisted in good nourishment, tonics and stimulants. Quinine in five-grain doses, from 13th to 21st, and smaller doses throughout.

A post-mortem held twenty-four hours after death revealed the following conditions: Cadaver greatly emaciated. Incision for the application of ligature to external iliac ununited, with superficial sloughing. Heart apparently normal. Lungs and kidneys greatly congested, and very dark color. Stomach and intestinal tract normal. After the contents of abdomen had been removed, an incision was carried through center of Poupart's ligament, parallel to femoral artery, extending nearly to the knee, when the external iliac and femoral arteries were dissected out from above downwards. A firm coagulum, fully an inch in length, had formed in femoral, below the seat of ligature; the inner coat of artery was found adherent for several lines. The vessel above the seat of ligature was gaping, and no evidence of a clot could be found. The cause of the secondary hæmorrhage from femoral was very apparent. Four unusually large muscular branches were given off from one to three lines above seat of ligature, allowing a free circulation of blood to the very seat of the ligature. This source of danger is not alluded to in any of the works on surgery at my command. The muscular branches were given off in the exact position where ligature of the femoral is recommended when the high operation is performed. From my experience in this case, if again called upon to ligate the femoral, would apply a ligature as close to the seat of hæmorrhage as practicable. My reasons for not ligating the popliteal were, the swollen condition of the limb in that region, as well as fear of a degenerate condition of the artery, as the anterior tibial would not bear a ligature, even when dissected back. The portion of the femoral and external iliac between the seat of the two ligatures was next examined, when the inner coat of the iliac was found firmly adherent, and could only be separated with difficulty. A firm coagulum was found in upper end of external iliac, extending nearly to the bifurcation of the common iliac. Slight adhesive inflammation had taken place between external iliac and surrounding tissues, the tissues at a distance of several lines from the artery presenting no abnormal appearance. The peritonæum presented a healthy appearance throughout. The epigastric and circumflex iliac arteries were given off immediately above Poupart's ligament, and directly opposite to each other. The specimen was carefully stuffed and dried, and has been preserved. I am inclined to believe that, had the patient been perfectly well at the time he sustained the original injury, he might ultimately have recovered.

FRACTURE OF PATELLA, WITH TREATMENT.

P. C., æt. 32 years, while playing "tack" upon the green, slipped, and in tiying to regain his equilibrium sustained, through sudden muscular contraction, a transverse fracture of the patella at junction of lower with middle third. The upper fragment was displaced fully an inch. The limb was placed in an extended position and cold applications made use of for fifteen hours. The injured knee had been swollen and painful for several years previous to the accident, duc to syphilitic rheumatism.

The dressing consisted of the usual single inclined plane extending for several inches beyond the heel, and raised about twelve inches at lower extremity; as this did not bring the fragments in apposition, an ordinary rubber bandage was applied in the following manner: The center of a strip twelve inches long and three inches wide was applied immediately above the upper fragment, when the rubber was stretched and either end fastened to side of splint with several tacks. The center of a second strip was carried in the same manner below the lower fragment and the ends fastened to sides of splints above. The fragments were at once drawn closer together, and at the end of twelve hours were in perfect apposition. This simple dressing was not disturbed more than twice during the entire treatment, extending over a period of six weeks. The tilting of the lower end of the upper fragment, usually so annoying, was entirely overcome by allowing the rubber band partially to overlap the upper fragment. The object of the lower rubber band is to fix the lower fragment. The elasticity of the rubber when thus applied makes a steady, equal traction, fixing the lower and gradually drawing the upper fragment downwards, as the muscular contraction of the rectus femoris is overcome. I claim the following points of superiority for this treatment:

1st. Simplicity, and easy application.

2d. Does not require as frequent renewals as non-elastic dressings.

3d. Makes continuous traction, bringing upper fragment downwards as the muscles relax.

4th. Fixes lower fragment, a point of considerable importance.

5th. Prevents the obstruction to the circulation which sometimes follows the application of the figure 8 bandage.

6th. Avoids the danger of severe inflammation sometimes encountered by the use of Malgaigne's hooks.

7th. The force applied can be regulated to a nicety, by increasing or diminishing the tension of the rubber bandage.

8th. Greater prospects of bony union, owing to closer apposition of fragments and immobility. One great objection to all non-elastic dressings, such as ordinary muslin bandages, is the fact that as the swelling decreases or the muscles relax the dressings become loosened and are of no further use, while the elastic dressings exercise continuous traction. The "Malgaigne hooks" I should be loth to make use of under any circumstances. Owing to the syphilitic complication, the iodides were administered throughout the treatment. At the end of six weeks the dressings were discontinued, the patient walked on crutches for about a week, but soon was able to walk as well as before the accident, with no increase in his limp. The union is bony throughout, and it is with difficulty that the seat of the fracture can be made out at present. The rubber bands are so easily applied, and fulfill the indications so well that, in a majority of cases, they need not be disturbed during the entire treatment.

RESECTION OF TIBIA AND FIBULA.

O. N., æt. 34 years, well-digger, fell a depth of forty feet into a well near Lima, Iowa, sustaining a compound comminuted fracture of tibia ; the fibula also was fractured about middle of leg. The leg was placed in a fracture box and extension applied for two weeks, when plaster paris dressings were applied for nearly five months. A very imperfect union having taken place, all dressings were discontinued. The leg at once began to bend backwards at seat of fracture, and, as the union became more firm, the leg could not be straightened. The deformity became so great that it was impossible for him to place his heel on the ground while in the erect position.

Being informed by the physicians who treated him that amputation of the deformed member was the only remedy left, he started for Chicago, hoping to find some relief in surgical interference short of an amputation. He was refused admission to the Cook County Hospital, Chicago, on account of the crowded condition of that institution, and was then sent to the Cook County Infirmary.

Upon examination, after admission, the bones at the seat of the fracture were found firmly united, a large provisional callus uniting the tibia and fibula into a common mass. The angle of deflection of the lower fragment was so great, that nothing but the extreme ends of the toes could be put to the floor while he maintained the erect position, and so painful was this position that he could not bear the weight of the leg upon the toes.

No history of syphilis was obtained, and his general health when admitted was good. Deciding upon a resection, with the assistance of Dr. Alex. Theummler, Assistant Superintendent of the Cook County Hospital for Insane, I performed the operation as follows: Carried an incision four inches in length along middle of anterior surface of tibia, directly over the seat of the callus, retracting both flaps as far as possible; next removing a triangular piece of bone three-fourths of an inch wide at base, extending completely through bones. The leg was then straightened, and two side splints well padded, extending from knee to an inch beyond the heel, were applied. The leg was suspended at an angle of fifteen degrees, and carbolized hot water dressings were applied continuously. The leg being perfectly straight and comfortable, the splints were allowed to remain for six weeks, when plaster Paris dressings were substituted for five weeks longer. When the splints were removed, slight motion could still be detected at the seat of the operation.

The plaster dressings had to be removed several times on account of erysipelas of the member, so severe that the life of the patient was threatened. When the plaster dressing was finally removed the union was firm. He at once began to use the member, walking with crutches for several weeks, gradually bearing more weight on the member, until not even a walking stick was needed to walk a distance of several miles. All pain had disappeared. The limb is one and one-half inches short.*

SYNOVITIS LEFT KNEE JOINT.

F. F., æt. 34 years; was admitted Dec. 27, 1881, with the following history: First noticed soreness and swelling twelve months previous to his admission. Was admitted to Cook County Hospital July 2. The excessive synovial fluid was remoned with aspirator and plaster paris cast applied and allowed to remain for two weeks, when it was removed, and extension substituted for two months. He was discharged as cured Dec. 14.

*Since the above was written, the patient passed through a severe attack of typhoid fever, recovering perfectly, and is able to use the limb nearly as well as ever.

After a few days of active exercise the pain and swelling returned, and he was transferred to this institution. When admitted the joint was greatly distended and very painful, the least motion causing great pain. He was at once sent to the hospital department, and extension applied by means of the ordinary adhesive plaster, rope and pulley appliance. The knee improved rapidly, but the extension was continued for nearly four months, when he was allowed to use the limb moderately. After a few days in the erect position the pain and swelling returned with increased intensity. Fly blisters and, later, pressure were resorted to, but the joint invariably became swollen and painful after a few days of moderate exercise. On Oct. 22 he applied to me for an operation to close a small cleft in palate, and when closely questioned admitted that he had had syphilis. Potassium iodide and hydrarg. bin-iodide were prescribed, and extension applied as before. At the end of three weeks all surgical appliances were discontinued and he was allowed to walk about the ward, and a few weeks later discharged as "recovered." He has been free from all symptoms of synovitis ever since, and works steadily in the asylum bakery, where he is compelled to stand upon the member several hours each day. The joint has been reduced to its normal size. The iodides are administered for several weeks at intervals of as many months. The interesting feature of the case is the undoubted specific character of the affection, as proven by the result of the treatment. Previous to the administration of the iodides the swelling and even most of the pain would disappear when the parts were placed at rest, only to return with increased severity after a few days of exercise of the member. After the iodides were administered the pain and swelling disappeared permanently after a very short interval of rest. I am satisfied that had the specific character of the affection been suspected, a permanent recovery would have followed his first admission to the hospital.

### MELANCHOLIA WITH SUICIDAL TENDENCIES.

On May 22, 1882, I was called to see J. W., an inmate of the infirmary employed in the asylum bakery. I found him lying in bed, covered with blood, and still bleeding freely from external jugular vein, which had been divided about the middle of its course. The wound had been inflicted with a shoemaker's knife ground to a sharp point. The wound was about two inches in length, very deep, and extending directly across the neck. The hæmorrhage was easily controlled by pressure, when the wound was closed, with several silk sutures. He was transferred to the infirmary hospital, where, on May 26, he made a very determined but unsuccessful effort to commit suicide by stabbing himself in nine places over abdominal region, with a pair of rusty scissors. Several of the wounds were quite deep, and it was feared had penetrated the peritoneal cavity. Owing to the position and character of the wounds, but little exploring was done, and it was not definitely ascertained whether the peritoneal cavity had been penetrated. Fortunately, the blades of the scissors were separated several inches; had they been closed, there can be no doubt that every stab would have entered the peritoneal cavity. On June 15 he was adjudged insane, and transferred to the Insane Asylum, where he made frequent efforts to commit suicide by suspension; also attempted to starve himself, and had to be fed with the stomach tube. His delusions were of a painful character. He imagined himself damned for committing the "unpardonable sin." The case is reported chiefly as an illustration of the persistency with which some insane patients strive to terminate their existence.

## Figures and Tables

**Figure f1:**
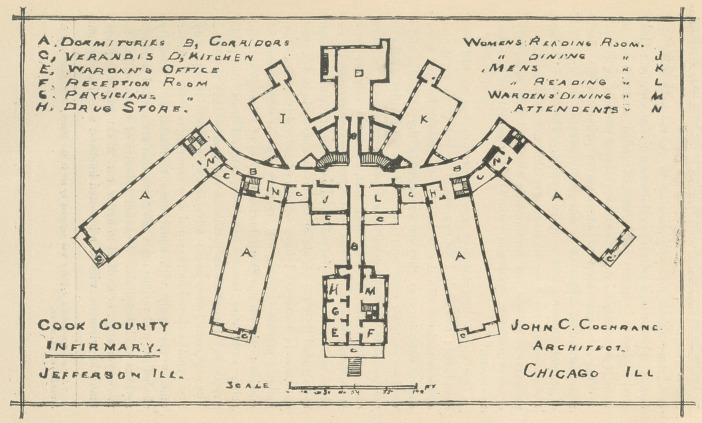


**Figure f2:**